# The Use of High Dose Letrozole in Ovulation Induction and Controlled Ovarian Hyperstimulation

**DOI:** 10.5402/2011/242864

**Published:** 2011-11-17

**Authors:** Elizabeth A. Pritts, Alexander K. Yuen, Shefaali Sharma, Robert Genisot, David L. Olive

**Affiliations:** Wisconsin Fertility Institute, 3146 Deming Way, Middleton, WI 53562, USA

## Abstract

Letrozole, an aromatase inhibitor, has been demonstrated to be effective as an ovulation induction and controlled ovarian hyperstimulation agent. However, dose administration has generally been limited to 5 days at 2.5 to 7.5 mg daily. We undertook a retrospective review of over 900 treatment cycles using letrozole in doses as high as 12.5 mg per day. Results indicate that such doses do indeed offer benefit to patients; in that there is increased follicular growth and a higher number of predicted ovulations with higher doses of the drug. However, increasing doses does not produce a detrimental effect upon endometrial thickness. High-dose letrozole may be of value in women who fail to respond adequately to lower doses. Furthermore, randomized trials are needed to determine whether high-dose letrozole might actually be optimal as a starting dose for certain treatment groups.

## 1. Introduction

In women undergoing ovulation induction for the treatment of oligoanovulation, clomiphene citrate has long been the initial drug of choice for first-line therapy [1]. The drug works primarily by competitively inhibiting the binding of estradiol to its receptor in the hypothalamus, thereby releasing the hypothalamus from negative inhibition and allowing increased release of follicle stimulating hormone (FSH) from the pituitary gland. This increase in FSH release enhances follicular growth, increasing the chances of ovulation. The drug has also proven useful for producing multiple ovulation in couples with unexplained infertility, male factor infertility, and other disorders where controlled ovarian hyperstimulation has been deemed of value.

While approved for use in the United States for more than 40 years, clomiphene has some significant limitations. First, only 75–80% of anovulatory women respond to the medication with appropriate follicular growth [1]. Furthermore, side effects of the drug can be psychologically difficult to endure (hot flashes and mood swings) and detrimental to fertility (impaired endometrial development and abnormal cervical secretions). The drug has a lengthy half-life, and adverse effects may be cumulative over time [2].

A class of drugs known as aromatase inhibitors also has the potential to enhance FSH release, not by the inhibiting estradiol-receptor interaction, but rather by inhibition of estradiol synthesis. One such inhibitor, letrozole, was approved for use in 1997 for the treatment of breast cancer. By 2001, it had been used in anovulatory women with great success, and at present the drug is extremely popular among physicians and patients in the treatment of both ovulation dysfunction and for controlled ovarian hyperstimulation: the drug has a half-life of only 45 hours, and side effects, while similar to those of clomiphene, are far milder and less frequent [3]. 

The original choice of dosing with letrozole was extrapolated from several studies performed on postmenopausal women being treated for breast cancer [4, 5]. Data derived from these patients suggested substantial inhibition of estradiol formation with doses of 2.5–5 mg daily. However, the application of these data to short-term use of the drug in reproductive age women is highly questionable. Nevertheless, clinical investigation of the drug in infertile women has been generally limited to 5 days of treatment at doses of 2.5–7.5 mg daily.

For several years, we have, in women felt to be suboptimally responding to established doses of letrozole, administered doses of the drug up to 12.5 mg daily. This manuscript was designed to examine the following questions: (1) is there a role for the use of high doses (greater than 7.5 mg daily) of letrozole in the treatment of chronic anovulatory patients?; (2) is there a role for the use of high-dose letrozole in controlled ovarian hyperstimulation?; (3) what effect does higher-dose letrozole have upon endometrial development?

## 2. Materials and Methods

This study is a retrospective cohort analysis with data extracted from our electronic medical record (eIVF, Practice Highway, Dallas). All patients treated with letrozole and intrauterine insemination at the Wisconsin Fertility Institute (Middleton, WI USA), from January, 2007, to December, 2009, were included in the study. Ages ranged from 23 to 47 years. All patients were administered 5 days of a fixed dose of letrozole beginning day 3 of their cycle; if the patient was anovulatory, medroxyprogesterone was administered to induce menses. Ultrasonography was performed on cycle day 11, and follicular number, follicular size, and endometrial thickness and pattern were determined; follicles were measured in two perpendicular dimensions and the mean value recorded, while endometrial thickness was measured at the point of greatest thickness.

Predicted ovulation number was calculated based upon the size of follicles on day 11, with a 1.7 mm per day adjusted increase until the day of triggering ovulation with human chorionic gonadotropin (hGG) [6]. Each extrapolated follicle size was then assigned a probability of ovulation taken from previously published data [7]. Summation of these probabilities yielded a single predicted ovulation number per cycle.

Descriptive statistics were calculated for all variables in an univariate manner. Multivariate linear and logistic regression analysis were performed to determine the relative importance of each predictor variable and its covariates. Terms remained in the equation as significant if *P* < 0.15. Eigen-value diagnostics were performed to identify potential problems with multicollinearity. One-way analysis of variance was performed as needed. Data were analyzed using the Statistical Package for the Social Sciences (SPSS) program.

## 3. Results

A total of 907 treatment cycles utilizing a five-day fixed dose of letrozole were identified in the two-year period. Of these, 41 (4.4%) were eliminated from the analysis due to abnormalities of medication administration/compliance or the absence of key information for one or more variables. Of the 866 remaining cycles, 33 were administered 5 mg daily, 80 were given 7.5 mg daily, 18 cycles utilized 10 mg daily, and 735 cycles used 12.5 mg daily. Results can be seen in Table 1. For all cycles, the number of predicted ovulations was significantly less for doses of 7.5 mg/day or less compared to doses of 10 mg/day or more (*P* < 0.001). This remained true when the data were filtered to include only first cycles on a given dose (*P* = 0.033). No significant differences were seen for pregnancy rates among doses.

### 3.1. High-Dose-Letrozole

As the 12.5 mg/day group was by far the largest, and due to the absence of literature addressing this dosage, univariate analysis was performed for this group. The overall mean for predicted ovulation number was 2.16 per cycle. The data were then filtered to remove nonindependent trials; analysis of only the first 12.5 mg dose for a given patient resulted in a mean predicted ovulation number of 1.91. Both data sets were analyzed for normality with no indication to reject the null hypothesis of the data being normally distributed (data not shown). Linear regression was then performed to determine if this value was altered significantly by age, BMI, or day-3 serum FSH level. Increasing BMI was significantly related to a decrease in predicted ovulation number for all 12.5 mg/day cycles (*P* = 0.002) and first 12.5 mg/day cycles (*P* < 0.001). None of the other variables had significant impact upon the outcome. 

The pregnancy rate for the 12.5 mg/day dose was 55/735 (7.5%). Logistic regression revealed that a diagnosis of anovulation resulted in a significantly higher pregnancy rate than other diagnoses (*P* = 0.003). Age, too, was significantly associated; increasing age lowered the pregnancy rate (*P* = 0.033).

The mean endometrial thickness on day 11 for all 12.5 mg/day cycles was 8.36 mm. All patients with more than one cycle at this dose were then identified, and the first and last cycle endometrial measurements were compared using a paired *t*-test. There was no difference in these measurements (initial thickness = 8.47, final thickness = 8.20; *P* > 0.1). Thus, there is no evidence that endometrial thickness will decrease with multiple cycles of high-dose letrozole.

### 3.2. Effect of Diagnosis on Outcome with High-Dose Letrozole

Since the treatment goal differs in women with ovulation dysfunction (1-2 ovulations) versus women undergoing controlled ovarian hyperstimulation with other diagnoses (2-3 ovulations or more), these groups were separated and compared (Table 2) [8]. 

For the anovulatory group treated with 12.5 mg/day, the mean number of predicted ovulations was 1.88. Regression analysis revealed BMI to have a negative effect upon predicted ovulation number (*P* = 0.049). The overall pregnancy rate in this group was 11%.

For the patients undergoing controlled ovarian hyperstimulation at this dose, the mean number of predicted ovulations was 2.03. This did not differ significantly from the anovulatory group. Regression analysis demonstrated BMI to have an inverse effect upon predicted ovulation number (*P* = 0.012) as did age (*P* = 0.062). The overall pregnancy rate in this group was 6%, significantly less than that of the patients with anovulatory cycles (*P* = 0.04).

### 3.3. Matched Pair Dose Comparison

A number of patients were treated with different doses of letrozole in multiple treatment cycles. In these matched pairs, lower doses (5, 7.5, and 10 mg/day) were compared to 12.5 mg/day (Table 3). Endometrial thickness did not vary significantly with any dose comparison, with 5mg dosing producing a scant 0.47 mm thicker endometrium than 12.5 mg daily. However, there was a significant difference in predicted ovulation number, with all three other dosing groups producing fewer ovulations than the 12.5 mg group, and statistical significance was reached comparing 7.5 mg versus 12.5 mg (*P* = 0.001).

## 4. Discussion

Letrozole has become an important tool in our armamentarium for treating infertility, yet surprisingly little effort has been devoted toward optimizing its effectiveness. The initial dosage schedule was extrapolated from that used with clomiphene, that is, 5 consecutive days beginning early in the follicular phase. The dosage range was chosen based upon estradiol suppression data from postmenopausal women. The latter subject is of concern in that there is hesitance on the part of physicians to explore higher doses of the drug. It may be that the use of higher doses than those commonly prescribed, especially in women who respond inadequately to standard doses, will allow more patients to remain on oral medications and not have to resort to gonadotropin therapy or in vitro fertilization.

Available evidence suggests a dose-response with letrozole, with higher doses producing more mature follicles and higher ovulation rates [9]. In the initial such study, 5mg daily produced a higher number of ovulations than 2.5mg [10]. A second study, comparing 2.5 mg, 5 mg, and 7.5 mg, found the number of mature follicles to be significantly greater as the dose increased (1.0, 1.4, and 3.4, resp.) [11]. 

This study suggests that there may be utility in increasing the dose even further, beyond 7.5 mg/day to as much as 12.5 mg/day. Predicted ovulation number was greater for increasing doses of the drug, and endometrial thickness was unaffected. Thus, when the patient's goal for number of predicted ovulations is not met with lower doses of the drug, it seems reasonable to explore their response to a dosage of 10–12.5 mg daily. 

Justification for not exceeding doses of 2.5–7.5 mg has been based on the concept that these doses reduce estradiol levels 88–98% [12]. However, these data, derived from postmenopausal breast cancer patients, may not be applicable to reproductive age women, especially those with elevated estrogen levels due to chronic anovulation and excessive BMI. Furthermore, at a dose of 2.5 mg daily, it takes 2–4 days for maximal suppression to occur [13]. Steady-state plasma levels do not occur for as long as 2 months [14]. Thus, there is reason to believe that higher-dose short-term administration may be more effective at inducing endogenous FSH release, resulting in greater follicular development.

In addition, estradiol suppression may not be the only effect of value. Letrozole has been noted to inhibit other aspects of the steroidogenic pathway, including a reduction in synthesis along the cortisol pathway [4]. Thus, intraovarian androgen accumulation may be disproportionately greater than the reduction in estrogen. Androgen has been well demonstrated in the primate to stimulate early follicular growth by augmenting follicular FSH expression and to stimulate endocrine and paracrine factors that synergize with FSH to promote folliculogenesis [15–21].

## 5. Summary

We have shown that letrozole, used in doses greater than those commonly employed, can produce enhanced follicular growth without detrimental effects upon the endometrium. Further study is clearly needed, including basic investigation into estradiol and androgen levels with these doses in reproductive age women. Nevertheless, we believe that high doses of this drug can and should be employed, particularly in women inadequately responsive to lower doses. In addition, we believe randomized trials comparing high-dose to low-dose administration would help determine the optimal starting dose for this medication in women of varying diagnoses.

## Figures and Tables

**Table:** (a) All cycles

Dose (mg)	*N*	Predicted ovulation number (95% CI)	Pregnancy rate (95% CI)
5	33	1.60 (1.32–1.89)	0.15 (0.02–0.28)
7.5	80	1.69 (1.50–1.89)	0.14 (0.06–0.21)
10	18	2.25 (1.17–2.79)	0.11 (0–0.27)
12.5	735	2.16 (2.09–2.23)	0.07 (0.06–0.09)

**Table:** (b) First cycle at indicated dose

Dose (mg)	*N*	Predicted ovulation number (95% CI)	Pregnancy rate (95% CI)
5	28	1.56 (1.25–1.87)	0.19 (0.03–0.35)
7.5	53	1.62 (1.39–1.85)	0.12 (0.03–0.21)
10	10	2.08 (1.30–2.87)	0
12.5	260	1.91 (1.80–2.02)	0.08 (0.05–0.12)

**Table 2:** Ovulations and pregnancy rate by diagnosis with letrozole 12.5 mg daily.

Diagnosis	Predicted ovulation number (95% CI)	Pregnancy rate (95% CI)
Ovulation dysfunction	1.88 (1.56–2.20)	0.11 (0.07–0.15)
Other	2.03 (1.82–2.24)	0.06 (0.04–0.08)

**Table 3:** Matched pair analysis.

Dose comparison	Endometrial thickness mean difference (mm) (95% CI)	*P* value	Predicted ovulation number mean difference (95% CI)	*P* value
5.0 versus 12.5	0.47 (−0.51–1.45)	0.33	−0.58 (−1.30–0.14)	0.11
7.5 versus 12.5	0.24 (−0.47–0.96)	0.49	−0.72 (−1.12–−0.32)	0.001
10.0 versus 12.5	−0.27 (−1.60–1.06)	0.65	−0.25 (−1.17–0.66)	0.54

## References

[1] Messinis IE (2005). Ovulation induction: a mini review. *Human Reproduction*.

[2] Glasier AF (1990). Clomiphene citrate. *Bailliere’s Clinical Obstetrics and Gynaecology*.

[3] Holzer H, Casper R, Tulandi T (2006). A new era in ovulation induction. *Fertility and Sterility*.

[4] Bisagni G, Cocconi G, Scaglione F, Fraschini F, Pfister C, Trunet PF (1996). Letrozole, a new oral non-steroidal aromastase inhibitor in treating postmenopausal patients with advanced breast cancer. A pilot study. *Annals of Oncology*.

[5] Buzdar AU, Robertson JFR, Eiermann W, Nabholtz JM (2002). An overview of the pharmacology and pharmacokinetics of the newer generation aromatase inhibitors anastrozole, letrozole, and exemestane. *Cancer*.

[6] Baerwald AR, Walker RA, Pierson RA (2009). Growth rates of ovarian follicles during natural menstrual cycles, oral contraception cycles, and ovarian stimulation cycles. *Fertility and Sterility*.

[7] Silverberg KM, Olive DL, Burns WN, Johnson JV, Groff TR, Schenken RS (1991). Follicular size at the time of human chorionic gonadotropin administration predicts ovulation outcome in human menopausal gonadotropin-stimulated cycles. *Fertility and Sterility*.

[8] Olive DL (1995). The role of gonadotropins in ovulation induction. *American Journal of Obstetrics and Gynecology*.

[9] Pritts EA (2010). Letrozole for ovulation induction and controlled ovarian hyperstimulation. *Current Opinion in Obstetrics and Gynecology*.

[10] Al-Fadhli R, Sylvestre C, Buckett W, Tan SL, Tulandi T (2006). A randomized trial of superovulation with two different doses of letrozole. *Fertility and Sterility*.

[11] Badawy AM, Metwally M, Fawzy M (2007). Randomized controlled trial of three doses of letrozole for ovulation induction in patients with unexplained infertility. *Reproductive BioMedicine Online*.

[12] Geisler J, King N, Dowsett M (1996). Influence of anastrozole (Arimidex), a selective, non-steroidal aromatase inhibitor, on in vivo aromatisation and plasma oestrogen levels in postmenopausal women with breast cancer. *British Journal of Cancer*.

[13] Demers LM, Lipton A, Harvey HA (1993). The efficacy of CGS 20267 in suppressing estrogen biosynthesis in patients with advanced stage breast cancer. *Journal of Steroid Biochemistry and Molecular Biology*.

[14] Bajetta E, Zilembo N, Dowsett M (1999). Double-blind, randomised, multicentre endocrine trial comparing two letrozole doses, in postmenopausal breast cancer patients. *European Journal of Cancer*.

[15] Weil SJ, Vendola K, Zhou J (1998). Androgen receptor gene expression in the primate ovary: cellular localization, regulation, and functional correlations. *Journal of Clinical Endocrinology and Metabolism*.

[16] Weil S, Vendola K, Zhou J, Bondy CA (1999). Androgen and follicle-stimulating hormone interactions in primate ovarian follicle development. *Journal of Clinical Endocrinology and Metabolism*.

[17] Vendola K, Zhou J, Wang J, Famuyiwa OA, Bievre M, Bondy CA (1999). Androgens promote oocyte insulin-like growth factor I expression and initiation of follicle development in the primate ovary. *Biology of Reproduction*.

[18] Vendola KA, Zhou J, Adesanya OO, Weil SJ, Bondy CA (1998). Androgens stimulate early stages of follicular growth in the primate ovary. *Journal of Clinical Investigation*.

[19] Adashi EY (1993). Intraovarian regulation: the proposed role of insulin-like growth factorsa. *Annals of the New York Academy of Sciences*.

[20] Giudice LC (1992). Insulin-like growth factors and ovarian follicular development. *Endocrine Research*.

[21] Yen SSC, Laughlin GA, Morales AJ (1993). Interface between extra- and intraovarian factors in polycystic ovarian syndrome. *Annals of the New York Academy of Sciences*.

